# Resilience of undergraduate health sciences students during COVID-19: An integrative review

**DOI:** 10.4102/hsag.v28i0.2331

**Published:** 2023-12-22

**Authors:** Elsie S. Janse van Rensburg

**Affiliations:** 1 Department of Health Studies, College of Human Sciences, University of South Africa, Pretoria, South Africa

**Keywords:** resilience, undergraduate, students, health sciences, COVID-19, pandemic, integrative review

## Abstract

**Background:**

COVID-19 pandemic placed pressure on global health systems, healthcare providers and undergraduate students in health sciences. Students experienced change in the teaching and learning as well as the clinical context resulting in increased stress levels. Resilience assisted students to adapt and develop competencies and effective coping mechanisms.

**Aim:**

The purpose of this integrative review is to identify resilience of undergraduate students in health sciences during the COVID-19 pandemic.

**Setting:**

Online platforms.

**Methods:**

An integrative review was conducted with keywords: resilience, undergraduate students, health sciences and COVID-19. Three different searches were conducted for the time frame 2020–2022 on the relevant electronic data bases with full text articles. A total of 1665 records were identified and 49 potentially relevant articles were identified. Screening resulted in 34 articles that were analysed using the John Hopkins critical appraisal criteria.

**Results:**

Four themes were identified: attributes of students’ resilience, aspects enhancing the development of resilience, aspects hindering the development of resilience and recommendations to cultivate resilience.

**Conclusion:**

Resilience is key to withstand the challenges in the global health system. The development of resilience in undergraduate health sciences students should be prioritised to ensure cognitive adaptability, effective coping skills and sufficient support.

**Contribution:**

These findings can assist higher educational institutions to improve their undergraduate health science programs to cultivate resilient health care professionals.

## Introduction

COVID-19 pandemic challenged health systems on a global scale (Ahmed et al. 2022; Ho et al. 2022). Health workers as well as students in health sciences were utilised as frontline workers. Lockdown restrictions resulted in limited access to face-to-face training at higher education institutions globally for students, including undergraduate health sciences students (Prieto et al. 2021).

This resulted in an andragogical shift to online platforms for teaching and learning (Shindjabuluka, Ashipala & Linkando 2022). The exposure as frontline workers to COVID-19 patients as well as the change in their learning environment increased the stress of undergraduate students in the health sciences domain (Abullais et al. 2022). A study conducted by Prieto et al. (2021) highlighted the fact that students had to adapt to an increased academic workload, challenges with the online learning platforms and chances in the teaching strategies.

Students felt overwhelmed, anxious and worried about how they would achieve their academic outcomes (Evans et al. 2021). Stress affected these students in different ways and for some increased the severity of pre-existing mental health challenges (Evans et al. 2021) while others demonstrated resilience that assisted them to navigate the demands during the COVID-19 storm effectively (Abullais et al. 2022).

## Aim

The aim of this integrative review is to identify resilience of undergraduate students in health sciences during the COVID-19 pandemic.

## Methods

An integrative review provides an all-inclusive exploration of a concept through a process of reviewing different designs (Knafl & Whittemore 2017). A systematic review analyses and combines experimental evidence according to a pre-set criteria to answer specific research questions (Lau & Kuziemsky 2017). The integrative review was chosen for this study as it includes qualitative and quantitative reviews and provided a comprehensive analysis on resilience within the specified context. An integrative review was used to identify, classify and analyse literature systematically (Toronto & Remington 2020) in order to address the aim.

### Search strategy

The researcher was supported by a subject librarian to identify and search databases for articles by using specific keywords. To ensure a thorough literature search, searches were conducted at three separate times during 2022 to ensure that all recent and relevant articles were included. The following electronic databases were included: EBSCOhost, Academic Search Premier, MEDLINE, CINAHL; Health Source: Nursing / Academic Edition, Africa-Wide Information, PubMed and ERIC. The inclusion criteria included the time frame between 2020 and 2022 and the keywords resilience, undergraduate students, health sciences and COVID-19 (Table 1). Only full-text articles were included. The researcher and subject librarian identified a total of 1665 bibliographic records during the initial search. Duplications were removed by the researcher and 49 potentially relevant articles were identified. Screening of the abstracts resulted in flagging 34 articles. The Johns Hopkins critical appraisal criteria (Johns Hopkins Evidence-Based Practice Model) was applied and resulted in 23 articles that were analysed (Table 2). The Johns Hopkins Nursing Evidence-Based Practice (JHNEBP) model provides a structured method to assess, combine and interpret evidence from different manuscripts (Sanluagn & Avant 2014).

**TABLE 1:** Criteria used during the search strategy with keywords.

Criteria	Include	Exclude
• Resilience	-	-
• Undergraduate students	-	-
• Health sciences	-	-
• COVID-19	-	-

**TABLE 2:** The critical appraisal process with the John Hopkins appraisal instrument for data extraction during the integrative review.

Number	Authors	Applicable population	Resilience theme	Strength of evidence	Quality of evidence	Included	Excluded
1	Virani et al. (2021)	Yes, undergraduate medical students	Online teaching & learning (Opportunities)	III	C	Yes	-
2	Aslam et al. (2021)	Yes, students of medicine, dentistry and allied health sciences	Online teaching & learning (Challenges); Mental health	I	A	Yes	-
3	GonÇalves et al. (2021)	No, all university students, not linked to Health Sciences	Not used	I	C	-	Yes
4	Coughenour et al. (2021)	No, all university students, not linked to Health Sciences	Not used	I	B	-	Yes
5	Brack et al. 2021	Yes, allied health students	Online teaching & learning (Opportunities)	III	C	Yes	-
6	Prieto et al. (2021)	Yes, dental students	Online teaching & learning (Opportunities & challenges); Mental health	III	A	Yes	-
7	Evans et al. (2021)	Yes, social work students	Online teaching & learning (Opportunities & challenges); Mental health, personal growth, resilience	I & III	A	Yes	-
8	Ezulike et al. (2021)	Yes, social work students	Online teaching & learning (Challenges); Mental health, financial impact, personal growth, resilience, coping mechanisms	III	A	Yes	-
9	Forycka et al. (2022)	Yes, medical students	Online teaching & learning (Challenges); Mental health	I	A	Yes	-
10	Gandhi et al. (2021)	Yes, nursing students	Mental health	I & III	A	Yes	-
11	Goodlet et al. 2022	No, pharmacy students in Doctor of Pharmacy programme	Mental health	I	B	-	Yes
12	Kerbage et al. (2021)	Yes, nursing students	Mental health, Coping strategies	I & III	B	Yes	-
13	Wang et al. (2021)	Yes, nursing students	Recommendations	II	C	Yes	-
14	Drach-Zahavy et al. (2022)	Yes, nursing students	Mental health	I	C	Yes	-
15	Kane et al. (2021)	Yes, nursing and midwifery students	Mental health, burnout	I & III	C	Yes	-
16	Kane et al. (2022)	Yes, nursing and midwifery students	Mental health, anxiety and professional development	I	C	Yes	-
17	Keener et al. (2021)	Yes, nursing students	Not used	III	C	-	Yes
18	Koob et al. (2021)	Yes, medical, nursing and social work students	Not used	III	C	-	Yes
19	Laher et al. (2021)	Yes, undergraduate psychology students	Mental health: anxiety and fear of contracting Covid themselves; Online teaching and learning challenges; coping skills, disturbance in personal life balance and recommendations	I & III	A	Yes	-
20	Leigh et al. (2020)	Yes, nursing students	Coping mechanism	III	C	Yes	-
21	Li Yu and Yang (2021)	No, college students. Does not specify	Not used	I	C	-	Yes
22	Maini et al. (2020)	Yes, medical students	Not used, limiting info	III	C	-	Yes
23	Mȕhlbauer et al. (2021)	Yes, medical students	Coping mechanisms: altruism	I	C	Yes	
24	Wallace et al. (2021)	Yes, nursing students	Online teaching & learning (Challenges & opportunities); Mental health, coping skills, recommendations	III	A	Yes	-
25	Smith, Urban & Wilson (2022)	Yes, nursing students	Not used	I	C	-	Yes
26	Gȍl and Erkin (2021)	Yes, nursing students	Mental health	I	C	Yes	
27	Labrague and Ballad (2021)	No, college students; does not specify	Not used	I	C		Yes
28	Pretorius (2021)	Yes, students from health sciences for physiotherapy, dietetics, psychology, occupational therapy and nursing	Mental health (negative effects – depression; positive effects – Resilience) Recommendations – Development of strengths (psychological preparedness) -Enhancing support (emotional support & awareness)	I	B	Yes	-
29	Rahman et al. (2021)	Yes, medical students	Mental health (negative effects: anxiety & depression; fear of covid: infecting others)	I	C	Yes	-
30	Razzak et al. (2022)	Yes, medical students	Not used	I	C	-	Yes
31	Renaud et al. (2021)	Yes, undergraduate and postgraduate medical students	Not used	I	C	-	Yes
32	Schlesselman et al. (2020)	Yes, pharmacy students	Online teaching and learning: Challenges (ineffective teaching skills; connectivity issues, screen tiredness) Disturbances in personal life balance (social isolation; lack of study space) Mental health: negative effect (increase existing symptoms) Recommendations (development of strengths: psychological preparedness)	I	C	Yes	-
33	Wang et al. (2021)	Yes, medical students	Mental health negative effects: Increased stress Online teaching and learning (challenges: increased stress) Recommendations (Creating learning and teaching spaces; enhancing support)	I	B	Yes	-
34	Yȕksel and Yilmaz (2022)	Yes, nursing students	Mental health – negative effects: increased stress Recommendations – Developing strengths (psychological preparedness)	I	B	Yes	-

Three steps were used by the researcher to identify relevant articles within the timeframe (2020–2022).

**FIGURE 1:**
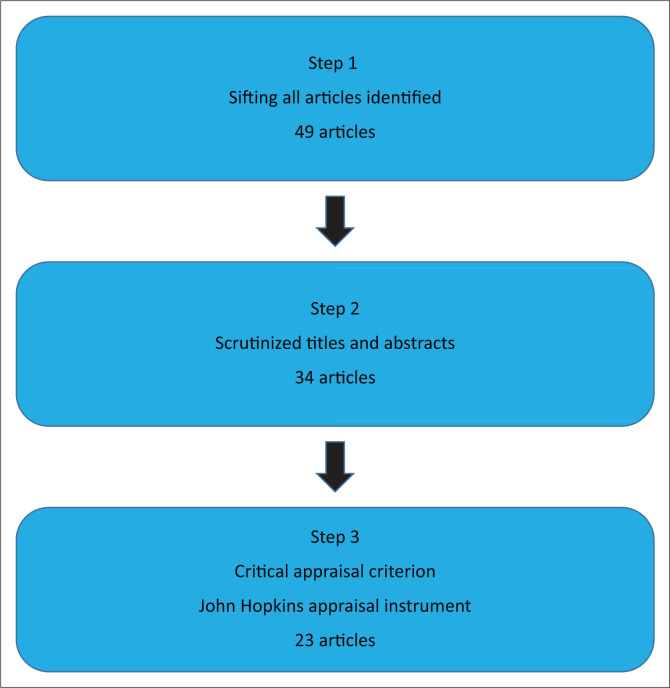
Steps in preparation of the data-extraction process.

**Step 1** (Figure 1), the researcher and subject librarian identified the appropriate articles based on the inclusion criteria and focused on resilience and undergraduate students in the health sciences disciplines during the COVID-19 pandemic. The researcher ensured that there were no duplications in this process. This step resulted in 49 potentially relevant articles that were applicable.

**Step 2** (Figure 1), the titles and abstracts of all 49 articles were analysed by the researcher to identify the most appropriate articles. Inclusion was based on resilience, undergraduate students, health sciences, COVID-19. This step led to identifying 34 articles.

**Step 3** (Figure 1), the researcher used the Johns Hopkins critical appraisal instrument to evaluate the quality of the 34 articles identified during step 2. The adapted Johns Hopkins appraisal instrument was used for the critical appraisal of the articles (Table 2). This step identified 23 articles.

### Critical appraisal: Data extraction

The Johns Hopkins appraisal instruments (JHNEBP Research Evidence Appraisal; Johns Hopkins University 2014) were used for the articles identified during Step 2 (Figure 1) to determine their appropriateness for inclusion in the integrative review. The critical appraisal process (Step 3 in Table 2) played a crucial role during this process. The inclusion criteria reflected the keywords (Table 1), population (resilience; undergraduate students; health sciences; COVID-19), strength and quality of evidence, strong study design and clear methodology. The quality of the scientific evidence was appraised according to the descriptions of the sample sizes; conclusions and recommendations based on a comprehensive literature review and findings.

The critical appraisal was applied as follows:

- Experimental (randomised controlled trial) or meta-analysis research
- Quasi-experimental research
- Non-experimental, qualitative and meta-synthesis research

The quality of the scientific evidence was rated according to:

A: High quality: reliable results with adequate sample size, conclusions and recommendations that were scientifically sound and supported with a literature review. The article had to have a sample size fitting with the methodology and reflected conclusions and recommendations based on a comprehensive literature review or findings.

B: Good quality: reasonable reliable results with an adequate sample size, fairly significant conclusions; reasonable consistent recommendations based on a moderately comprehensive literature review. The article had to have a sample size fitting with the methodology and have made some conclusions and recommendations based on a partial literature review.

C: Low quality: inconsistent results from an inadequate sample from where no conclusions were drawn.

### Results of the critical appraisal

From the 34 publications reviewed, 23 were included after the critical appraisal process was completed.

### Rigour

A subject librarian assisted with literature searches by using specified keywords and search engines to enhance accuracy. The literature search was conducted during three different times in 2022 to look for articles that fit the inclusion criteria. The critical appraisal was applied to 34 articles and 11 were excluded because of poor quality of evidence, inadequate conclusions and recommendations or findings irrelevant to the topic under study. The sample size of 23 was based on data saturation where the themes were recurring, and no new information was added. A co-coder ensured neutrality. Thematic analysis was applied following Tesch’s protocol as described in Creswell (2014). Themes, categories and subcategories were identified and described in Table 3.

**TABLE 3:** Themes, categories, and sub-categories of the integrative review on resilience of undergraduate students in health sciences during COVID-19.

Themes	Categories	Sub-categories
1. Attributes of students’ resilience		1.1.1 Defining of resilience invulnerability (Concalves et al. 2021:5) embedded in a socio-ecological perspective (Drach-Zahavy et al. 2021:111) the ability of a person to navigate their way to resources that sustain well-being Individual, family and community level (Drach-Zahavy et al. 2021:111; 115) 1.1.2 Self-efficacy and self-determination … I taught classes with a few of my peers (Wallace et al. 2021:616) … we [*as students*] felt helpless not knowing what to do … over time, I have become more self-reliant (Vázquez-Calatayud et al. 2021:126) …[*students*] gained confidence (Ghandi et al. 2021:8) 1.1.3 Adaptability during remote learning … after we do our classes, we get together and do our own classes … (Wallace et al. 2021:616) … [*The COVID-19 pandemic*] prompted many students to enhance their resourcefulness and creativity (Wallace et al. 2021:616) … [*students*] stayed connected to their studies by actively seeking out opportunities via online platforms such as zoom … to communicate with teachers and peers (Kerbage et al. 2021:1412) 1.1.4 Resilience is protective of students’ well-being … resilience partially mediates the relationship between hopelessness and depression… (Pretorius 2021:275) … higher levels of resilience … protect [*students*] their well-being (Drach-Zahavy et al. 2021:116) … As personal, relational, organizational and national resilience is augmented…students are able to maintain their well-being (Drach-Zahavy et al. 2021:117)
2. Aspects enhancing the development of resilience	2.1. Cognitive adaptability cultivating students’ resilience	2.1.1 Creating opportunities for personal and professional growth … guided self-reflection and mentor support… can yield opportunities for positive personal growth (Goodlet et al. 2022:36) Everything I have witnessed, whether it be good or bad, will influence my future practice as a student and nurse. I will reflect on the things that I have experienced and when I feel drained and defeated through life, work or when I’m struggling with an assignment, these things will remind me that I am strong and that, no matter how tough things get, it will all be worth it for both myself and my patients (Leigh et al. 2020:788) Though, physically exhausting and emotionally draining, I have developed strong emotional resilience (Kane et al. 2022:99) 2.1.2 Contributing to society It was like a very rapid evolution … We went from the fear of being away from my family, friends, university … to the frustration and helplessness of not knowing how to help… to finding our place as professionals and … feeling useful (Vázquez-Calatayud et al. 2021:126) I felt a social obligation as I am nearly qualified and … in a place to help my community (Kane et al. 2022:97) It’s so amazing to hear the positive stories of communities coming together to support our healthcare and key workers [*in the Netherlands*] (Leigh et al. 2020:789) 2.1.3 Fostering healthy relationships with peers and self During the transition to virtual learning, the mantra of ‘we are all in this together’ (Goodlet et al. 2022:34) … [*COVID*] pushed me out of my comfort zone and allowed me to ‘show up’ in ways I never knew I could (Evans et al. 2021:779) … [*I developed the*] ability to take time for self-care (Wallace et al. 2021:616) 2.1.4 Adversity as an opportunity to learn and acquire new skills [*Social Work*] students to receive Telehealth Training (Evans et al. 2021:780) … [*Students*] learned how to work in disaster management (Ghandi et al. 2021:8) I felt this time would give me a unique experience (Kane et al. 2022:99) 2.1.5 Viewing the positive side of challenges … contentment is the key… the gift of life … [*to*] find happiness (Ezulike et al. 2021:10) … do the best with what you got (Wallace et al. 2021:616) Staying positive in times of uncertainty (Kerbage et al. 2021:1411)
	2.2. Coping strategies cultivating students’ resilience	2.2.1 Emotion-focused coping Managing negative emotions Each time I feel down emotionally, I play the piano… Social media was a good coping strategy (Ezulike et al. 2021:10) … whenever I have free time, I tried to exercise as much as I can (Wallace et al. 2021:616) … Religion and spirituality… (Ezulike et al. 2021:10) 2.2.2 Problem-focused coping Active actions to cope or solve problems … [*Students*] Counselled themselves… learned a new skill (Ezulike et al. 2021:10) Debriefing…developing routines (Kerbage et al. 2021:1411) … shared reflections… (Leigh et al. 2020:789) 2.2.3 Altruism and satisfaction to be of help to others … assisting other people kept [*students*] them busy and took their minds off the current situation (Ezulike et al. 2021:10) Self-satisfaction in life-saving process and in service to the needy… (Ghandi et al. 2021:9) I have been trained for this. It is my responsibility (Vázquez-Calatayud et al. 2021:127) 2.3.1 Institutional support … university [*was seen*] as supportive … provision of data, counselling services …support offered by lecturers and tutors (Laher et al. 2021:224) … There was a very good atmosphere in the team and a great deal of collaboration …. (Vázquez-Calatayud et al. 2021:127) … [*supervisor*] checking on my wellbeing … guiding me (Kane et al. 2022:99)
	2.3. Support cultivating students’ resilience	2.3.2 Peer support … we felt like we were going through this together. Nobody was alone, and we had the support that we needed (Wallace et al. 2021:615) Having colleagues close by… knowing that the other person will understand you because they are in the same situation as you … if I needed to cry, I wept… (Vázquez-Calatayud et al. 2021:127) 2.3.3 Social and professional support My family members provided little financial support. They also provided huge emotional support by encouraging me not to give up hope (Ezulike et al. 2021:9) I have a regular Zoom meeting with my church group to stay connected (Kerbage et al. 2021:1411) I’m also seeing a therapist on a regular basis … it also helped me (Wallace et al. 2021:616)
3. Aspects hindering the development of resilience	3.1 Challenges in cultivating students’ resilience	3.1.1 Coping challenges … some students used negative coping strategies such as avoidance. While such a coping strategy proves effective in the short term, it may be ineffective in the long term (Ezulike et al. 2021:14) … self-distraction coping strategy… (Laher et al. 2021:221) Low resilience was correlated with more severe burnout, poor well-being, reduced motivation, and higher usage of stimulants… (Forycka et al. 2022:10)
4. Recommendations to cultivate students’ resilience	4.1 Recommendations to cultivate students’ resilience	4.1.1 Capacity building to cultivate resilience and mental health … universities to implement programmes to educate students… increase awareness regarding mental health… signs and symptoms of hopelessness and depression (Pretorius 2021:275) … online counselling sessions, workshops on coping and stress… (Aslam et al. 2021:85) Students must be given the tools to manage their anxiety (Yüksel & Yilmaz 2022:81) 4.1.2 Enhancing institutional support (academic & emotional) … the lecturers in the Department, reach out to the students…at different time points, to know how well they are faring…assure [*them*] they are not alone… (Ezulike et al. 2021:14) … foster online community building, become adept in teaching in an online environment… promote self-care to cultivate resilience (Wallace et al. 2021:617) … maintain transparency about the [*educational*] goals … Share your own experiences…offer flexible structures…provide ongoing feedback (Schlesselman et al. 2020:680) 4.1.3 Providing psychological support for students More access to counselling services and self-help material was recommended (Laher et al. 2021:224) Allow student to determine how much they can withstand … Ask students what makes them feel safe… Communicate often… Invite conversation … Address students by name … Ask students how they are doing (Schlesselman et al. 2020:680) … [*it was recommended that*] students with moderate anxiety should be followed up after graduation and supported to ensure that they do not experience more severe anxiety while working in clinical settings (Yüksel & Yilmaz 2022:81)

## Ethical considerations

This article followed all ethical standards for research without direct contact with human or animal subjects.

## Results

There were four themes reflected in the findings: attributes of students’ resilience, aspects enhancing the development of resilience, aspects hindering the development of resilience and recommendations to cultivate students’ resilience. Table 3 indicates the thematic analysis applied to the data.

## Discussion

This integrative review aimed to identify resilience of undergraduate students in health sciences during the COVID-19 pandemic.

Data from 23 articles reflected four themes: (1) attributes of students’ resilience, (2) aspects enhancing the development of resilience, (3) aspects hindering the development of resilience and (4) recommendations to cultivate students’ resilience.

### Attributes of students’ resilience

The findings indicated that students displayed attributes that enhanced their resilience (Drach-Zahavy et al. 2021; Gandhi et al. 2021).

The following sub-categories were identified during data analysis: defining resilience, self-efficacy and self-determination, adaptability and resilience is protective of students’ well-being.

#### Defining resilience

According to Gonçalves et al. (2021), the origin of the term resilience is found in the 1970s when American and British researchers used the term ‘invulnerability’ for people who stayed well despite exposure to difficult times. From a psychological perspective, resilience is used to describe people’s ability to face challenges and struggles in a positive manner (Chen & Bonanno 2020 in Gonçalves et al. 2021). These challenges can include adaptability to change (Killgore et al. 2020 in Gonçalves et al. 2021). Resilience is also defined by Drach-Zahavv et al. (2021) from a socio-ecological viewpoint as the ability to predict, prepare, survive and adjust to turbulent environments by using available resources. These resources can be collective on an individual, family or community level.

#### Self-efficacy and self-determination

Students indicated that the COVID-19 pandemic strengthened their independence as their loved ones were not near to assist them with challenges. In a study by Wallace et al. (2021), students formed study groups on their own where they simulated and practised nursing skills. Their independence grew as they discovered their ‘own strength’ and developed their nursing competencies. Students also felt more confident (Ghandi et al. 2021) and self-reliant as reflected by a study:

At first, we were afraid of not having our loved ones nearby… then we felt helpless not knowing what to do, … over time, I have become more self-reliant. (Vázquez-Calatayud et al. 2021:126)

#### Adaptability during remote learning

The COVID-19 pandemic provided the opportunity for students to be resourceful and innovative during online learning. One student in a study conducted by (Wallace et al. 2021:616) indicated ‘I taught classes with a few of my peers’. Another student said:

I had to find ways to be creative, to learn how to do a lot of the nursing tasks. And so my creativity expanded. I made my own tools. I made my own Foley catheters … and I did a bunch of stuff with teddy bears … (Wallace et al. 2021:616)

According to a study conducted by (Kerbage et al. 2021:1409), adaptability was improved by developing daily routines and ‘staying connected’ with friends, family and their studies through utilising online platforms such as Zoom to collaborate with their peers and educators.

#### Resilience is protective of students’ well-being

Pretorius (2021) highlights the balance between hopelessness and depression and points out that resilience lowers the incidence of hopelessness resulting in depression. The link between resilience and well-being is supported by Drach-Zahavy et al. (2021) that indicate nursing students with resilience are less prone to suffer from intense stress.

### Aspects enhancing the development of resilience

This theme had three categories: cognitive adaptability; coping strategies cultivating students’ resilience and support cultivating students’ resilience.

#### Cognitive adaptability cultivating students’ resilience

This category has five sub-categories that include:

1. creating opportunities for personal and professional growth;
2. adversity as an opportunity to contribute to society;
3. fostering healthy relationships with peers and self;
4. adversity as an opportunity to learn and acquire new skills; and
5. viewing the positive side of challenges.

**Creating opportunities for personal and professional growth:** A study conducted by Goodlet et al. (2022) with pharmacy students revealed that self-reflection and mentor support during challenges can facilitate personal development during COVID-19. In another study by Leigh et al. (2020:788), a nursing student said:

‘Everything I have witnessed, whether it be good or bad, will influence my future practice as a student and nurse. I will reflect on the things that I have experienced and when I feel drained and defeated through life, work or when I’m struggling with an assignment, these things will remind me that I am strong and that, no matter how tough things get, it will all be worth it for both myself and my patients.’ (student 1, nurse)

This view was supported by a nursing student in a study by Kane et al. (2022) that pointed out that though physically exhausting and emotionally draining, they have developed strong emotional resilience’. Students from social work reflected on their academic challenges during the pandemic as follows:

‘I have learned how resilient and flexible I am. I am not a fan of distance learning, but having it forced upon me, made me face some fears and overcome them.’ (student 2, nurse)

**Adversity as an opportunity to contribute to society:** Students in a study conducted in Nigeria by Ezulike, Okoye and Ekoh (2021) reflected on how the COVID-19 pandemic motivated them to ‘make the world a better place’. They felt that their minds were broadened to think not only of themselves but other healthcare workers and communities. The nursing students appreciated the opportunity that the COVID-19 pandemic provided for them to join other health care workers as frontline workers (Leigh et al. 2020). Student nurses in a study conducted by Vázquez-Calatayud et al. (2021) experienced the COVID-19 pandemic as a chance to improve the lives of patients and their families through health promotion.

**Fostering healthy relationships with peers and self:** The challenges of the COVID-19 pandemic created a platform where relationships with peers could be nurtured and developed (Kerbage et al. 2021; Wallace et al. 2021). A student in a study by Goodlet et al. (2022:34) relates.

During the transition to virtual learning, the mantra of ‘we are all in this together’ was emphasized … as well as the need to be flexible and compassionate while navigating a new reality. (p. 34)

One of the social work students talked about how difficult it was during the COVID-19 pandemic but also said:

I can honestly say that strangely it has brought me closer to family and friends and pushed me out of my comfort zone and allowed me to ‘show up’ in ways I never knew I could. (Evans et al. 2021:779)

**Adversity as an opportunity to learn and acquire new skills:** Students from different disciplines reflected on the new skills they learned including disaster management (Ghandi et al. 2021; Kane et al. 2022). Social work students indicated in a study conducted by Evans et al. (2021) that they were able to learn how to utilise telehealth to support the health needs of diverse communities.

**Viewing the positive side of challenges:** Students highlighted the need to stay positive by stating:

… contentment is the key… the gift of life … [*to*] find happiness. (Ezulilike 2021:10)

… do the best with what you got. (Wallace et al. 2021:616)

Staying positive in times of uncertainty. (Kerbage et al. 2021:1411)

#### Coping strategies cultivating students’ resilience

Literature indicated that coping strategies influence students’ resilience (Ezulike et al. 2021; Ghandi et al. 2021; Kerbage et al. 2021; Leigh et al. 2020; Wallace et al. 2021). There are three sub-categories identified for this category, namely (1) emotion-focuses coping, (2) problem-focused coping and (3) altruism.

**Emotion-focused coping:** Some students indicated that they cope by managing their negative emotions by relaxing, exercising and religion (Ghandi et al. 2021; Kerbage et al. 2021). Relaxation included playing games or piano or listening to music. Students mentioned the following:

Each time I feel down emotionally, I play the piano … Social media was a good coping strategy. (Ezulilike 2021:10)

… whenever I have free time, I tried to exercise as much as I can. (Wallace et al. 2021)

… Religion and spirituality … (Ezulilike 2021:10)

**Problem-focused coping:** Students from different disciplines indicated that they actively try to solve their problems. They reflected on the following skills:

… [*Students*] Counselled themselves … learned a new skill. (Ezulilike 2021:11)

Debriefing … setting boundaries between work and study…developing routines. (Kerbage et al. 2021:1412)

… shared reflections … (Leigh et al. 2020:789)

**Altruism:** Students stated that altruism refers to helping others. They reflected on how altruism enhanced their resilience as follows:

… assisting other people kept [students] them busy and took their minds off the current situation. (Ezulilike 2021:10)

I realized the power of our profession, that we hold people’s lives in our hands … I grasped the importance of the nursing profession, of accompanying patients in good and bad times … I have been trained for this. It is my responsibility. (Vázquez-Calatayud et al. 2021:127)

Self-satisfaction in life-saving process and in service to the needy… (Ghandi et al. 2021)

#### Support cultivating students’ resilience

This category has three sub-categories that include (1) institutional support, (2) peer support and (3) social and professional support.

**Institutional support:** Institutional support was viewed by students as support from the university in terms of data provision, counselling services and support from lecturers (Evans et al. 2021; Laher et al. 2021; Vázquez-Calatayud et al. 2021).

**Peer support:** Peer support was perceived by students from different disciplines as ‘virtual get-togethers to maintain relationships with classmates’ (Goodlet et al. 2022). In a study conducted by Wallace et al. (2021), peer support was reflected on by nursing students that described forming their own study groups through social media platforms to enhance peer interactions. One student said … we felt like we were going through this together. Nobody was alone, and we had the support that we needed (Wallace et al. 2021).

Another student indicated:

Having colleagues close by… knowing that the other person will understand you because they are in the same situation as you … if I needed to cry, I wept … (Vázquez-Calatayud et al. 2021:127)

**Social and professional support:** Participants from the studies (Ezulike et al. 2021; Kerbage et al. 2021; Wallace et al. 2021) related social support as being provided by their families and professional support by therapists and pastors.

### Aspects hindering the development of resilience

This theme highlighted the challenges in cultivating students’ resilience by exploring their coping challenges. These challenges referred to avoidance (Ezulike et al. 2021) and self-distracting (Laher et al. 2021). A study conducted by Forycka et al. (2022) added ‘Low resilience was correlated with more severe burnout, poor well-being, reduced motivation, and higher usage of stimulants…’

### Recommendations to cultivate students’ resilience

This theme had two sub-categories: (1) capacity building to cultivate resilience and (2) enhancing institutional support on academic and emotional levels.

#### Capacity building to cultivate resilience and mental health

Capacity building was seen as increasing awareness though programmes, online counselling sessions and workshops (Aslam et al. 2021; Pretorius 2021; Yȕksel & Yilmaz 2022).

#### Enhancing institutional support

Institutional support was classified on an academic and emotional level as follows:

… the lecturers in the Department, reach out to the students … to know how well they are faring … assure [*them*] they are not alone … (Ezulilike 2021:12)

… foster online community building, become adept in teaching in an online environment … promote self-care to cultivate resilience. (Wallace et al. 2021:617)

… maintain transparency about the [*educational*] goals…Share your own experiences … offer flexible structures … provide ongoing feedback. (Schlesselman, Cain & DiVall 2020:680)

Students from different disciplines emphasised the need for access to counselling services and self-help materials (Laher et al. 2021). Follow-up of students after graduation to support them while they are working in clinical settings was also indicated by a study conducted by Yȕksel and Yilmaz (2022).

## Contribution

This review contributes to cultivating resilience of undergraduate students in health sciences during their training to prepare them for global pandemics and disasters. Preparation in the form of capacity building and support is needed to equip these students to withstand any challenging context in their professional career, enhancing the global health systems.

The role of student support on an institutional level was highlighted to develop student resilience. Student support can include face-to-face or online counselling sessions as well as workshops, webinars and self-help materials. Capacity building can focus on emotional awareness and development of effective coping skills, cognitive adaptability, as well as personal and professional development. Growth and development can be facilitated through online short courses with assessments and reflections to monitor progress.

## Conclusion

This review achieved the initial aim by indicating that undergraduate students in health sciences demonstrated resilience during the COVID storm. The participants included in this review represented the global context and included different disciplines: pharmacy, social work, medical, dentistry, psychology and nursing students.

Findings highlighted attributes of students’ resilience as self-efficacy, self-determination, adaptability, personal and professional growth. Aspects enhancing the development of resilience included cognitive adaptability, effective coping strategies (emotion-focused, problem-focused and altruism) and support (institutional, peer, social and professional). Aspects hindering the development of resilience referred to ineffective coping skills (avoidance and self-distraction).

Recommendations are made to cultivate resilience as part of the undergraduate curriculum on health sciences to empower these students to bring hope and healing during times of turmoil.

## References

[CIT0001] Abullais, S.S., Khan, A.A., AlQahtani, S.A., Zuhayr, A.Z.A., Parveen, S., Alassiri, A.S. et al., 2022, ‘Coronavirus disease (COVID-19) associated anxiety, fear and preparedness among healthcare students at University teaching hospital in KSA’, *Psychology Research and Behavior Management* 15, 875–885. 10.2147/PRBM.S347313 35431586 PMC9012232

[CIT0002] Ahmed, T., Dumka, N., Bhagat, D., Hannah, E. & Kotwal, A., 2022, ‘Effect on essential health services during COVID-19 at the primary level in India’, *Journal of Family Medicine and Primary Care* 11(9), 5423–5429. 10.4103/jfmpc.jfmpc_390_22 PMC9731057 36505582

[CIT0003] Aslam, K., Zaidi, S.H.R., Arooj, M. & Sethi, A., 2021, ‘COVID-19 pandemic: How stressed the students and faculty are?’, *Journal of Medical Sciences* 29(3), 83–86. https://doi.org/10.52764.jms.21.29.3.4

[CIT0004] Brack, P., Bramley, A., Downie, S., Gardener, M., Leo, J., Sturt, R., et al., 2021, ‘Riding the waves: lessons learnt from Victoria’s COVID-19 pandemic response for maintaining effective allied health student education and clinical placements’, *Australian Health Review* 45, 683–689. 10.1071/AH21145 34847339

[CIT0005] Concalves, M.P., Freires, L.A., Tavares, J.E.T., Vilar, R. & Gouveia, V.V., 2021, ‘Fear of COVID and trait anxiety: Mediation of resilience in university students’, *Psicologia:Teoria e Pratica* 23(1), 1–16. https://doi:10.5935/1980-6906/ePTPC1913996

[CIT0006] Coughenour, C., Gakh, M., Pharr, J.R., Bungum, T. & Jalene, S., 2021, ‘Changes in depression and physical activity among college students on a diverse campus after a COVID-19 stay-at-home order’, *Journal of Community Health* 46, 758–766. 10.1007/s10900-020-00918-5 33165765 PMC7649574

[CIT0007] Creswell, J.W., 2014, *Research Design: Qualitative, quantitative and Mixed Methods Approaches*, 4th edn., Sage, Thousand Oaks, CA.

[CIT0008] Drach-Zahavy, A., Goldblatt, H., Admi, H., Blau, A., Ohana, I. & Itzhaki, M., 2022, ‘A multi-level examination of nursing students’ resilience in the face of COVID-19 outbreak: A cross-sectional design’, *Journal of Advanced Nursing* 78(1), 109–120. 10.1111/jan.14951 34212420 PMC8446960

[CIT0009] Evans, E.J., Reed, S.C., Caler, K. & Nam, K., 2021, ‘Social work students’ experiences during the COVID-19 pandemic: Challenges and themes of resilience’, *Journal of Social Work Education* 57(4), 771–783. 10.1080/10437797.2021.1957740

[CIT0010] Ezulike, C.D., Okoye, U.O. & Ekoh, P.C., 2021, ‘Social work undergraduates students and COVID-19 experiences in Nigeria’, *Qualitative Social Work* 21(5), 880–896 10.1177/14733250211029705 PMC9434194 36068916

[CIT0011] Forycka, J., Pawlowicz-Szlarska, E., Burczyńska, Cegielska, N., Harendarz, K. & Nowicki, M., 2022, ‘Polish medical students facing the pandemic – Assessment of resilience, well-being and burnout in the COVID-19 era’, *PLoS One* 17(1), e0261652. 10.1371/journal.pone.0261652 35073318 PMC8786167

[CIT0012] Gandhi, S., Sahu, M., Govindan, R., Nattala, P., Gandhi, S., Sudhir, P.M. et al., 2021, ‘Psychological preparedness for pandemic (COVID-19) management: Perceptions of nurses and nursing students in India’, *PLoS One* 16(8), e0255772. 10.1371/journal.pone.0255772 34388177 PMC8362956

[CIT0013] Gol, L. & Erkin, O., 2021, ‘Mental status of nursing students assessed using the general health questionnaire during the COVID-19 pandemic in Turkey’, *Perspectives in Psychiatric Care* 57(4), 1712. 10.1111/ppc.12740 33616202 PMC8013214

[CIT0014] GonÇalves, M.P., Freires, L.A., Tavares, J.E.T., Vilar, R. & Gouveia, V.V., 2021, ‘Fear of COVID and trait anxiety: Mediation of resilience in university students’, *Psicologia: Teoria e Practica* 23(1), 1–16. 10.5935/1980-6906/ePTPC1913996

[CIT0015] Goodlet, K.J., Raney, E., Buckley, K., Afolabi, T., Davis, L., Fettkether, R.M. et al., 2022, ‘Impact of the COVID-19 pandemic on the emotional intelligence of student pharmacist leaders’, *American Journal of Pharmaceutical Education* 86(1), 32–36. 10.5688/ajpe8519 PMC8787171 34301541

[CIT0016] Ho, L.S., Bertone, M.P., Mansour, W., Masaka, C. & Kakesa, J., 2022, ‘Health system resilience during COVID-19 understanding SHR service adaptation in North Kivu’, *Reproductive Health* 19, 135. 10.1186/s12978-022-01443-5 35668397 PMC9169445

[CIT0017] Kane, C., Rintakorpi, E., Wareing, M. & Hewson, D., 2021, ‘The psychological effects of working in the NHS during a pandemic on final-year students: Part 1’, *British Journal of Nursing* 30(22), 1303–1307. https://di.org/10.12968/bjon.2021.30.22.1303 34889683 10.12968/bjon.2021.30.22.1303

[CIT0018] Kane, C., Wareing, M. & Rintakorpi, E., 2022, ‘The psychological effects of working in the NHS during a pandemic on final-year students: Part 2’, *British Journal of Nursing* 31(2), 96–100. https://di.org/10.12968/bjon.2022.31.2.96 35094541 10.12968/bjon.2022.31.2.96

[CIT0019] Keener, T.A., Hall, K., Wang, K., Hulsey, T. & Piamjariyakul, U., 2021, ‘Relationship of quality of life, resilience, and associated factors among nursing faculty during COVID-19’, *Nurse Educator* 46(1), 17–22. 10.1097/NNE.0000000000000969 32941307

[CIT0020] Kerbage, S.H., Garvey, L., Willetts, G. & Olasoji, M., 2021, ‘Undergraduate nursing students’ resilience, challenges, and supports during corona virus pandemic’, *International Journal of Mental Health Nursing* 30(1), 1407–1416. 10.1111/inm.12896 34109714

[CIT0021] Knafl, K. & Whittemore, R., 2017, ‘Top 10 tips for undertaking synthesis research’, *Research in Nursing & Health* 40(3), 189–193. 10.1002/nur.21790 28267870

[CIT0022] Koob, C., Schropfer, K., Coenen, M., Kus, S. & Schmidt, N., 2021, ‘Factors influencing study engagement during the COVID-19 pandemic: A cross-sectional study among health and social professions students’, *PLoS One* 16(7), e0255191. 10.1371/journal.pone.0255191 34314450 PMC8315536

[CIT0023] Labrague, L.J. & Ballad, C.A., 2021, ‘Lockdown fatigue among college students during the COVID-19 pandemic: Predictive role of personal resilience, coping behaviors, and health’, *Perspectives in Psychiatric Care* 57(4), 1905–1912. 10.1111/ppc.12765 33728666 PMC8251079

[CIT0024] Laher, S., Bain, K., Bemath, N., de Andrade, V. & Hassem, T., 2021, ‘Undergraduate psychology student experiences during COVID-19: Challenges encountered and lessons learnt’, *South African Journal of Psychology* 51(2), 215–228. 10.1177/0081246321995095

[CIT0025] Lau, F. & Kuziemsky, C., 2017, *Handbook of eHealth evaluation: An evidence-based approach*, University of Victoria, Victoria. 29431951

[CIT0026] Leigh, J., Bolton, M., Cain, K., Harrison, N., Bolton, N.Y. & Ratcliffe, S., 2020, ‘Student experiences of nursing on the front line during the COVID-19 pandemic’, *British Journal of Nursing* 29(13), 788–789. 10.12968/bjon.2020.29.13.788 32649251

[CIT0027] Li, W., Gillies, R., He, M., Wu, C., Lui, S., Gong, Z. et al., 2021, ‘Barriers and facilitators to online medical and nursing education during the COVID-19 pandemic: Perspectives from international students from low- and middle-income countries and their teaching staff’, *Human Resources For Health* 19(1), 64. 10.1111/jan.14960 33980228 PMC8114664

[CIT0028] Maini, A., Saravanan, Y., Singh, T.A. & Fyfe, M., 2020, ‘Coaching skills for medical education in a VUCA world’, *Medical Teacher* 42(11), 1308–9. 10.1080/0142159X.2020.1788713 32657666

[CIT0029] Muhlbauer, L., Huber, J., Fischer, M.R., Berberat, P.O. & Gartmeier, M., 2021, ‘Medical students’ engagement in the context of the SARS-CoV-2 pandemic: The influence of psychological factors on readiness to volunteer’, *GMS Journal of Medical Education* 38(6). 10.3205/zma001506 PMC8493846 34651068

[CIT0030] Pretorius, T., 2021, ‘Depression among health care students in the time of COVID-19: The mediating role of resilience in the hopelessness-depression relationship’, *South African Journal of Psychology* 51(2), 269–278. 10.1177/0081246321994452

[CIT0031] Prieto, D., Tricio, J., Cāceres, F., Param, F., Melēndez, C., Vāsquez, P. et al., 2021, ‘Academics’ and students’ experiences in a Chilean dental school during the COVID-19 pandemic: A qualitative study’, *European Journal Dental Education* 25(4), 689–697. 10.1111/eje.12647 33368901

[CIT0032] Rahman, S.T., Khan, M.M. & Islam, S.T.A., 2021, ‘Online education system in Bangladesh during COVID-19 pandemic’, *Creative Education* 12(2), 441–452. 10.4236/ce.2021.122031

[CIT0033] Razzak, A.R., Al-Shaibani, T. & Naguib, Y., 2022, ‘Do students effectively learn physiology through distance online instruction? Medical students’ perceptions and academic performance’, *Advances in Physiology Education* 46(1), 65–70. https://doi.org/10/1152/advan.00098.2021 34817296 10.1152/advan.00098.2021

[CIT0034] Renaud, M.C., Vatier, C., Simon-Tillaux, N., Hertig, A. & Jeru, I., 2021, ‘Lessons from the impact of COVID-19 on medical educational continuity and practices’, *Advances in Physiology Education* 45(2), 390–398. 10.1152/advan.00243.2020 33961515 PMC8384569

[CIT0035] Sanluagn, C.S. & Avant, K., 2014, ‘A critical synthesis of literature review on the selected John Hopkins nursing evidence based practice model’, *International Proceedings of Social and Behavioral Sciences* 2(1), 131–141, viewed from http://researchgate.net/profile/Chayapha-Sanluang/publication/333668002_A_Critical_Synthesis_of_Literature_Review_on_the_Selected_John_Hopkins_Nursing_Evidence_Based_Practice_Model/links/5ef51807a6fdcc4ca430fde7/.

[CIT0036] Schlesselman, L.S., Cain, J. & DiVall, M., 2020, ‘The COVID-19 pandemic across the academy. Improving and restoring the well-being and resilience of pharmacy students during a pandemic’, *American Journal of Pharmaceutical Education* 84(6), 677–682. 10.5688/ajpe8144 PMC7334351 32665720

[CIT0037] Shindjabuluka, R.N., Ashipala, D.O. & Linkando, G.N., 2022, ‘COVID-19 as an enabler for enhancing online learning and teaching skills for nurse educators at the University of Namibia: Prospects and challenges’, *HealthSA Gesondheid* 27, a1727. 10.4102/hsag.v27i0.1727 PMC8905325 35281284

[CIT0038] Smith, J.G., Urban, R.W. & Wilson, S.T., 2022, ‘Association of stress, resilience, and nursing student incivility during COVID-19’, *Nursing Forum* 57, 374–381. 10.1111/nuf.12694 35032050

[CIT0039] Toronto, C.E. & Remington, R., 2020, *A step-by-step guide to conducting an integrative review*, Springer International Publishing.

[CIT0040] Vázquez-Calatayud, M., Rumeu-Casares, C., Olana-Lizarraga, M. & Martínez, E.R., 2021, ‘Nursing students’ experience of providing frontline COVID-19 support: A qualitative study’, *Nursing and Health Sciences* 24(1), 123–131. 10.1111/nhs.12902 34761512 PMC8662254

[CIT0041] Virani, S., Handuleh, J.I., Pereira-Sanchez, V. & Wolde-Giorgis, D.F., 2021, ‘Teaching psychiatry in a low-income country during the COVID-19 pandemic: A hybrid collaborative psychiatry course’, *Asia-Pacific Psychiatry* 13(4), e12503. 10.1111/appy.12503 34967115

[CIT0042] Wallace, S., Schuler, M.S., Kaulback, M., Hunt, K. & Baker, M., 2021, ‘Nursing student experiences of remote learning during the COVID-19 pandemic’, *Nursing Forum* 56(3), 612–618. 10.1111/nuf.12568 33728660 PMC8250930

[CIT0043] Wang, J., Lui, W., Zhang, Y., Xie, S. & Yang, B., 2021, ‘Perceived stress among chinese medical students engaging in online learning in light of COVID-19’, *Psychology Research and Behavior Management* 14, 549–562. 10.2147/PRBM.S308497 34017205 PMC8131094

[CIT0044] Yȕksel, A. & Yilmaz, E.B., 2022, ‘Nursing student attitudes toward nursing profession and their state anxiety level during COVID-19 outbreak’, *Journal of Psychiatric Nursing* 13(1), 76–82. 10.14744/phd.2021.39205

